# Advancing Alzheimer’s Disease Modelling by Developing a Refined Biomimetic Brain Microenvironment for Facilitating High-Throughput Screening of Pharmacological Treatment Strategies

**DOI:** 10.3390/ijms26010241

**Published:** 2024-12-30

**Authors:** Nuraqila Mohd Murshid, Nur Fatin Nabilah Mohd Sahardi, Suzana Makpol

**Affiliations:** 1 Department of Biochemistry, Faculty of Medicine, Level 17 Preclinical Building, Universiti Kebangsaan Malaysia, Jalan Yaacob Latif, Bandar Tun Razak, Cheras, Kuala Lumpur 56000, Malaysia; nuraqilamohdmurshid@ukm.edu.my; 2 Secretariat of Research and Innovation, Faculty of Medicine, Universiti Kebangsaan Malaysia, Jalan Yaacob Latif, Bandar Tun Razak, Cheras, Kuala Lumpur 56000, Malaysia; nurfatinnabilah@ukm.edu.my

**Keywords:** Alzheimer’s disease, biomimetic brain, beta-amyloid plaque, tau proteins

## Abstract

Alzheimer’s disease (AD) poses a significant worldwide health challenge, requiring novel approaches for improved models and treatment development. This comprehensive review emphasises the systematic development and improvement of a biomimetic brain environment to address the shortcomings of existing AD models and enhance the efficiency of screening potential drug treatments. We identify drawbacks in traditional models and emphasise the necessity for more physiologically accurate systems through an in-depth analysis of current literature. This review aims to study the development of an advanced AD model that accurately replicates key AD pathophysiological aspects using cutting-edge biomaterials and microenvironment design. Incorporating biomolecular elements like Tau proteins and beta-amyloid (Aβ) plaques improve the accuracy of illustrating disease mechanisms. The expected results involve creating a solid foundation for high-throughput screening with enhanced scalability, translational significance, and the possibility of speeding up drug discovery. Thus, this review fills the gaps in AD modelling and shows potential for creating precise and efficient drug treatments for AD.

## 1. Introduction

As Malaysia’s population ages, the number of older adults is growing rapidly, exceeding earlier predictions. By 2050, over 15% of the population is projected to be over 65. This trend suggests a higher risk of Alzheimer’s disease (AD), which affects people differently [1]. The incidence of AD is rising as the world’s population ages, highlighting the urgent need for effective treatments and preventative measures. China has seen a surge in Alzheimer’s cases, becoming the global leader [2]. Moreover, while typically associated with old age, Alzheimer’s is now affecting younger individuals in China. The disease’s duration varies greatly, ranging from 2 to 3 years to 20 years for different individuals [3]. However, Alzheimer’s disease is not limited to these regions. With the proportion of older adults rapidly increasing worldwide, more than 55 million people are currently affected by AD or other common forms of dementia, such as frontotemporal degeneration, cerebrovascular disease, hippocampal sclerosis, Lewy body disease, and even Parkinson’s disease, according to the World Health Organization. This figure is expected to triple by 2050 [4]. Notably, AD prevalence varies across regions, with higher rates observed in North America and Europe compared to Asia and Africa. These differences are likely influenced by factors such as life expectancy, lifestyle, and access to healthcare.

Therefore, the urgent need to find a cure for AD highlights the importance of creating new, scientifically based models. In the past, researchers have mainly concentrated on understanding AD by studying protein misfolding and aggregation [5,6,7,8]. It is no surprise that much research has been conducted to investigate the relationship between amyloid-beta (Aβ) plaques and tau tangles and how they contribute to the onset of AD. This link has been thoroughly investigated, and the results indicate that tau tangles and Aβ plaques play a role in developing this devastating condition [9,10,11,12,13,14]. Gradually, the buildup of Aβ aggregates initiates a number of intricate processes that affect tau proteins, interfere with glial cell and neuron function, and eventually cause dementia symptoms [15,16,17,18]. Lifestyle choices and genetics both influence the risk and mechanisms of AD. Comorbidities such as type 2 diabetes mellitus [19,20,21,22,23], stroke [24,25,26], hypercholesterolemia [27,28,29,30], heart disease [31,32,33,34], traumatic brain injury [35,36,37,38], obesity [39,40,41,42], and lifestyle or physical inactivity [43,44], as well as age-related cognitive decline [45,46], are contributors to AD pathogenesis. Pharmacological efforts aimed at targeting anti-amyloid or anti-protein misfolding strategies have, unfortunately, not produced a conclusive disease-modifying treatment for AD [47].

Over the past 25 years, numerous drugs targeting Aβ in AD trials, including five anti-Aβ antibodies (bapineuzumab [48,49], solanezumab [50,51], crenezumab [52], ponezumab [53,54], and gantenerumab [55]), have failed to demonstrate clinical effectiveness. A notable addition to this lineup was Aducanumab (Aduhelm^®^), introduced in June 2021 [56]. Although Aducanumab effectively reduced amyloid plaques, it did not yield improved clinical outcomes for Alzheimer’s patients [57,58,59]. One major concern surrounding Aducanumab was the high incidence of side effects, particularly amyloid-related imaging abnormalities (ARIAs). These side effects, common and dose-dependent with human monoclonal antibodies, affected about one-third of participants in Aducanumab trials. While one trial indicated that individuals receiving Aduhelm experienced less Alzheimer’s deterioration than those on a placebo, a second trial demonstrated similar worsening in both Aduhelm and placebo groups. Consequently, the European Medicines Agency (EMA) rejected Aducanumab in December 2021, highlighting the difficulties in finding effective treatments for AD [60]. As a result, a variety of molecular theories have been investigated, including metal dyshomeostatis, oxidative stress, mitochondriopathy, gliopathy, and synaptopathy. Despite these studies, a conclusive therapy breakthrough is still elusive [61].

However, the emergence of these novel hypotheses and mechanisms has often been perceived as antagonistic and even competitive. Definitive proclamations have been made, dismissing the amyloid hypothesis as invalid and advocating for its substitution. There is a debate about whether amyloid-beta is a leading player or just one part of the disease process [62,63]. However, studies on how brain cells react to Aβ, and genetic links show a strong connection between Aβ and Alzheimer’s. Rather than rejecting Aβ or any other proposed explanation, creating a new AD model that combines different ideas into one comprehensive explanation is essential. This inclusive approach is vital to finding a cure for Alzheimer’s. As a result, researchers use a range of models to explain the complex processes of AD and develop novel treatments. This review aims to address gaps in AD modelling and demonstrates the potential for developing precise and effective drug treatments.

## 2. Biomolecular Components Relevant to AD

**Figure 1:**
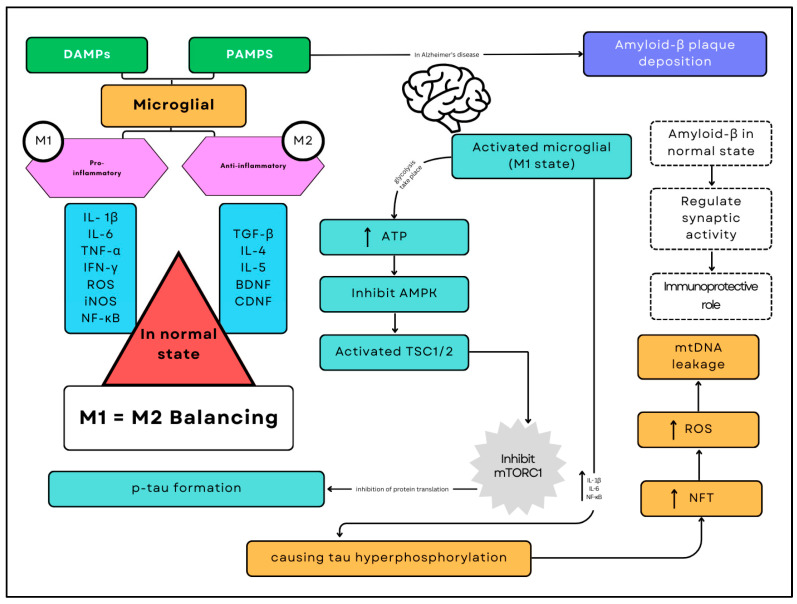
Alzheimer’s disease pathogenesis. The pathogenesis of Alzheimer’s disease involves a complex interplay of immune and metabolic processes mediated by microglia, the brain’s resident immune cells. The deposition of amyloid-beta (Aβ) plaques serves as a critical trigger in this cascade. As Aβ plaques accumulate, they activate microglia through Damage-Associated Molecular Patterns (DAMPs) and Pathogen-Associated Molecular Patterns (PAMPs), shifting them predominantly into the pro-inflammatory M1 state. This activation results in increased glycolysis and higher ATP consumption, which inhibits AMP-activated protein kinase (AMPK) and activates the Tuberous Sclerosis Complex proteins (TSC1/2), ultimately leading to the inhibition of the mechanistic Target of Rapamycin Complex 1 (mTORC1).

The key player in AD pathogenesis derives from the intricate interaction between sirtuins and the mechanistic target of rapamycin (mTOR) [64]. A vital regulator of cellular processes, mTOR plays a crucial role in the production of Aβ peptides, the stimulation of tau hyperphosphorylation, and protein synthesis [65,66] (Figure 1). Misregulation is worsened by mTOR-triggered neuroinflammation [67]. SIRT1 and other sirtuins are involved in cellular stress responses and can impact the removal of Aβ by activating autophagy and deacetylating tau [68,69]. There is complexity in the relationship between mTOR and sirtuins since SIRT1 suppresses mTOR signalling, which influences the delicate balance between cell survival and death [70]. Furthermore, mTOR and sirtuins are essential for regulating mitochondrial activity and energy metabolism, both of which are critical to the complex nature of AD pathogenesis [71,72]. Understanding these interconnected pathways offers valuable information on potential therapeutic approaches to address Aβ deposition, tau pathology, and neurodegeneration in AD.

Moreover, Aβ peptides, particularly Aβ42 and Aβ40, also play a critical role in the development of AD. Amyloid precursor protein (APP) is the source of these peptides, which are produced by the coordinated action of β- and γ-secretase [73,74]. They have a tendency to assemble and form insoluble plaque that is intricately present in the tapestry of AD-affected brains. Similar to recognisable landmarks, these plaques guide the path towards the disease’s diagnosis. Aβ peptides cause a series of disruptions to neuronal function in addition to their architectural presence [75,76]. Aβ oligomers, the intermediate characters in this unfolding drama, disrupt the delicate balance of synaptic function. This disruption impairs the release of neurotransmitters and the long-term stimulation process, which is essential for memory formation [77,78].

Furthermore, neuroinflammation contributes significantly to the complex interplay between the central nervous system and the immune system in the pathophysiology of AD [79,80]. In the immunological response of the brain, microglia and astrocytes are essential for coordinating inflammatory responses that are directly related to the development of AD [81]. Activated microglia serve as protectors of immune surveillance and break down abnormal protein aggregates like Aβ plaques through phagocytosis [82,83,84]. However, unexpected effects might occur when there is an imbalance in the intricate interactions between pro-inflammatory cytokines, chemokines, and reactive oxygen species. Chronic pro-inflammatory conditions have an adverse effect on neuronal structure where microglial persistence transitions to a more aggressive state, escalating neuroinflammation and impeding the progression of the disease [85,86].

Consequently, astrocytes, which were generally considered to have supportive roles, have been shown to have a dual nature in neuroinflammation by participating in the complex coordination of neurological processes. During chronic inflammation, these astrocytes transform into reactive states that release inflammatory mediators while withholding the crucial support necessary for neuronal survival [87,88]. This astrocytic dysfunction contributes to AD progression by breaking down the blood-brain barrier (BBB). The infiltration of peripheral immune cells significantly increases the inflammatory response when the BBB malfunctions [89,90,91]. This relentless neuroinflammatory environment plays a significant role as a main character in the progression of AD, intricately woven into its initiation and progression. This dynamic process further challenges the resilience of neurons, testing their ability to withstand the evolving complexity that characterises the landscape of AD.

Moreover, tau proteins change the AD environment since they are essential for maintaining the integrity of microtubules in the nervous system [92]. The transformed tau proteins undergo abnormal phosphorylation, leading to the development of neurofibrillary tangles (NFTs). Tau, typically a stabilising force for microtubules, loses its grip due to genetic predispositions and environmental influences, thus forming insoluble NFTs [93]. NFTs cast a shadow and have a negative impact on the neuronal landscape by disrupting microtubules and hindering intracellular transport [94]. The essential movement of cellular components along neuronal processes falters, affecting the distribution of nutrients and signalling molecules. Synaptic function, an integral chapter in the story of cognitive decline, faces adversity [95,96]. Tau aggregates establish a cascade reaction that triggers off pathways leading to oxidative stress, inflammation, and cell death [97]. The unfolding of tau proteins across interconnected brain regions represents a tragic propagation of neurodegeneration, contributing to the gradual and widespread demise of neurons in the Alzheimer’s narrative [98].

Furthermore, mitochondria have emerged as a key player in the pathophysiology of AD [99]. Their primary function, elucidated as the cellular powerhouse, undergoes a transformation, contributing substantively to the slow death of nerve cells [99]. Mitochondrial dysfunction emerges as a subtle subplot, triggering a decline in adenosine triphosphate (ATP) generation-a vital energy source for neurons [100]. This intricate interplay within the cellular milieu underscores the significance of mitochondrial dysfunction as a pivotal aspect in understanding AD progression. Moreover, in mitochondrial dysfunction, oxidative stress emerges as a pivotal antagonist, playing a role in the cascading events of AD development [100]. Impaired electron transport elevates reactive oxygen species (ROS), unleashing oxidative harm upon the neuronal landscape. The interaction between mitochondrial dysfunction and oxidative stress becomes a self-perpetuating cycle that hastens the deterioration of neuronal function [101]. Once heralded as protectors, the mitochondria become unwitting contributors to the unfolding tragedy of AD.

Alzheimer’s narrative is characterised by an early chapter on synaptic dysfunction, which contributes significantly to the cognitive decline witnessed by individuals with the condition [102]. Synapses, the communication points between neurons, face an early demise, reflecting the progression of cognitive decline [96]. Neurotransmitter release encounters interference and synaptic plasticity, a dynamic element in the cognitive storyline. Aβ aggregates, tau pathology, and the inflammatory subplot collaboratively disrupt the normal process of synapses [96]. Aβ oligomers, the elusive characters in this synaptic drama, interfere with synaptic transmission, binding to receptors and disrupting signalling pathways [77]. Tau pathology becomes a formidable force, disrupting microtubules and hindering the transport of synaptic vesicles.

When synaptic dysfunction occurs in AD, the BBB acts as a protector, controlling the flow of substances between the blood and the brain, thereby forming an environment for the unfolding narrative [103]. The breakdown of the BBB becomes a pivotal plot point, allowing the infiltration of detrimental substances into the brain’s sacred territory-from inflammatory molecules to toxins. The compromised barrier integrity escalates the neuroinflammatory environment within the central nervous system [104]. Immune cells from the periphery become unwitting characters in this unfolding drama, intensifying the inflammatory reaction [104]. The understanding of BBB alterations offers a glimpse into the early chapters of AD, unravelling potential therapeutic targets and opening new avenues for intervention in the ongoing narrative of AD.

A balance between M1 (pro-inflammatory) and M2 (anti-inflammatory) microglial states is essential for maintaining normal brain function in a healthy brain. However, in Alzheimer’s disease, the predominance of the M1 state disrupts this balance. The sustained activation of M1 microglia promotes hyperphosphorylation of tau protein, forming neurofibrillary tangles (NFTs) and increasing the release of pro-inflammatory cytokines such as interleukin-1β (IL-1β), interleukin-6 (IL-6), and nuclear factor kappa B (NF-κB). This inflammatory environment exacerbates neuronal damage, generating reactive oxygen species (ROS) and causing leakage of mitochondrial DNA (mtDNA). These pathological processes accelerate neurodegeneration and cognitive decline, characterising the progression of Alzheimer’s disease.

## 3. Existing Animal Models for AD

Many models have appeared in this context, each providing distinct perspectives on AD pathogenesis. The models vary from cellular and molecular systems to animal models, each created to mimic particular aspects of AD pathology and aid in investigating underlying mechanisms. The complex interplay of genetic, molecular, and environmental factors that impact AD onset and course may be better understood by researchers using these models. Existing models for AD encompass a wide range of approaches, from transgenic mouse models to computational and data-driven models. Understanding β-amyloid toxicity and the involvement of various β-amyloid species has been a key focus of studies on AD pathophysiology utilising transgenic mouse models including 5xFAD, APP3, PDAPP, Tg2576, P301S, and 3xTg-AD rodent models [105,106,107]. These models have provided a significant understanding of the pathogenic pathways of AD, particularly related to β-amyloid oligomers.

### 3.1. PDAPP

The first AD model that displayed the deposit of Aβ was the PDAPP mouse model. The Indiana mutation (V717F) in the human amyloid precursor protein (APP) gene is expressed in PDAPP mice [108]. This gene is regulated by the platelet-derived growth factor (PDGF)-β promoter [109]. Age-related increases in gliosis correspond to the formation of Aβ in the brain’s cortex, which starts to occur between the ages of six and nine months old. Moreover, at eight months of age, the hippocampus dentate gyrus molecular layer’s synaptic and dendritic densities start to decline. Cognitive deficits, particularly memory impairments, become evident in 3 months and worsen with age.

### 3.2. 5xFAD

Since the 5xFAD transgenic mouse model of AD consists of five mutations linked to familial AD, it is often used for studying the late stages of AD diseases [110]. They involve two mutations in the presenilin-1 (PS1) gene (L386V and M14L) and three in the APP gene (Swedish K670N/M671L, Florida I716V, and London V717I). According to Oakley et al. [111], developing APP/PS1 double transgenic mice that co-express 5xFAD causes an increase in Aβ42 synthesis and deposition, which causes amyloid plaques to rapidly and significantly accumulate in the brain. Another study found that β-amyloid plaque was also accumulated in the spinal cord of a 5xFAD mouse model in both the grey and white matter [112]. Girard et al. [113] found that 5xFAD mice have the highest corticolimbic Aβ compared to another model. Cognitive deficiencies in AD, which are characterised by the presence of gliosis and amyloid plaque, were linked to the presence of Aβ in 5xFAD mice, particularly in the frontal cortex of the brain. This frontal cortex has a crucial role in learning abilities, which could be an early indicator of AD-related cognitive decline [113]. Poon et al. [114] explore the relationship between cognitive impairment, neuronal loss, and amyloid deposition in the 5xFAD transgenic mouse. The hippocampus and entorhinal regions of female 5xFAD mice show significantly higher levels of amyloid deposition than those of male mice, highlighting the sex-related differences in AD. Keszycki, Rodriguez, Dunn, Locci, Orellana, Haupfear, Dominguez, Fisher, and Dong [105] indicated that 5xFAD mice exhibit apathy-like behaviours that worsen as they age. Amyloid plaques and soluble Aβ42 levels in the hippocampus and prefrontal cortex were linked to this apathy-like behaviour.

Additionally, the rise in β-site APP-cleaving enzyme 1 (BACE1) was associated with the overproduction of Aβ in AD [115]. The BACE1 causes the accumulation of β-cleaved C-terminal fragment C99 and full-length amyloid precursor protein (fl-APP) in the mitochondria, leading to mitochondrial dysfunction and cognitive impairment [116]. The pathology of neurodegeneration in AD is also related to alterations in astrocyte and oligodendrocyte gap junction expression in the 5xFAD mouse spinal cord [117]. Meanwhile, Hüttenrauch displayed that neprilysin (NEP) deficiency in the 5xFAD mouse modifies AD’s neuropathological and behavioural phenotype. NEP is recognised as an enzyme that breaks down Aβ in AD progression. The decrease in NEP level in 5xFAD contributed to increased Aβ accumulation. The effect of short-term and long-term exposure to volatile anaesthetics on neuropathological and behavioural systems was also observed in 5xFAD mice [118]. Prolonged exposure to a volatile anaesthetic significantly enhanced Aβ deposition in the hippocampal CA1 and CA2 regions and activated glial cells in the amygdala.

Meanwhile, Giesers and Wirths [119] demonstrated that, in 5xFAD mice, a notable reduction in calretinin-positive interneurons was observed in the hippocampal CA1 and CA2/3, while parvalbumin-positive interneurons were reduced throughout the hippocampal, particularly in the dentate gyrus. These interneurons may be lost in the 5xFAD mice model due to the presence of extracellular amyloid plaques. The administration of orphan nuclear receptor Nurr1 (NR4A2) in a 5xFAD mouse model regulates the AD pathophysiology by reducing Aβ plaque deposition, neuronal loss, microgliosis, and impairment of adult hippocampus neurogenesis [120].

### 3.3. APP23

The APP23 mouse model encodes the APP with the Swedish mutation (KM670/671NL), which contributes to the increased production of Aβ peptides [121]. Spatial memory deficit in the AD APP23 transgenic mouse model occurs before the formation of plaques and the subsequent increase in plaque-associated amyloid-β1-42 peptides [122]. The Aβ deposition plaques were observed in the cerebral cortex, thalamus, olfactory nucleus, hippocampus, and putamen of the APP23 mouse model [123]. In addition, there was selective neuronal death in the brain regions of APP23 mice [124]. Lefterov et al. [125] showed that ABCa1 is necessary for memory impairment in old APP23 mice, which are probably affected by the quantity of accumulated Aβ oligomers in the hippocampus. Along with the formation of amyloid plaque, the APP23 transgenic mouse model also showed loss of pyramidal neurons in the hippocampus CA1 area, with the activated microglia linked to amyloid plaques and hyperphosphorylation of tau protein [121].

Boncristiano et al. [126] displayed that there was a cholinergic modification in AD. The cholinergic deficit in the cortex of the APP23 mice is induced by amyloid accumulation and impairment of cholinergic basal forebrain neurons. However, AD is not facilitated by disturbance of the basal cholinergic forebrain system. Amyloid plaque formation and amyloid-associated microglial activation in the brain of APP23 transgenic mice are stimulated by neuron-derived beta precursor protein (βPP) [127]. Aβ causes some commissural neuron types to gradually degenerate, primarily affecting the most complicated dendritic structure. This degeneration begins with Aβ deposits appearing and worsens as Aβ deposits spread to other brain regions [128]. In another study, Yue et al. [129] demonstrated that oestrogen -deficient APP23 mice exhibited increased Aβ deposition and impaired Aβ degradation by microglia, indicating that oestrogen reduction in the brain may significantly influence the progression of AD neuropathology. However, Aβ deposits were decreased in the absence of enzyme tissue transglutaminase (TG2) [106]. This TG2 was found abundantly in the human brain. It is crucial for post-translation modification in Aβ, producing covalently linked, stable, and toxic Aβ aggregates.

### 3.4. Tg2576

A transgene with the Swedish mutation (KM670/671NL) in the APP gene is present in Tg2576 mice. In Tg2576 transgenic mice, the increased Aβ may be associated with synaptic dysfunction even without synapse loss [130]. Meanwhile, Kalback et al. [131] demonstrated that the increase in soluble Aβ levels with age is accompanied by relatively minimal vascular amyloid deposition. King and Arendash [132] evaluated the role of synaptophysin immunoreactivity (SYN-IR) in the hippocampus and neocortex regions of Tg2576 transgenic mice. This SYN-IR is known as a marker for synaptic terminals. King and Arendash [132] displayed that maintained SYN-IR during ageing is related to impaired synaptic function, contributing to cognitive deficits in AD. Another study, which utilised Tg2576 transgenic mice to explore β-amyloid-mediated inflammation in AD, discovered that the neocortex and hippocampus of Tg2576 mice models had diffuse and senile β-amyloid plaques [133]. The early β-amyloid exposure induces both pro- and anti-inflammatory mechanisms by upregulating TGF-β, IL-1β, and IL-10 in adjacent reactive astrocytes. Senile plaques disrupt cortical cytoarchitecture, leading to a gradual loss of antioxidant capacity [134].

### 3.5. PS01S

The PS01S mouse model integrates APP and presenilin-1 (PS-1) gene mutation related to familial AD. Tau aggregation initially occurs in the PS01S transgenic mouse model’s cerebral cortex and hippocampus [135], followed by the appearance of neurofibrillary tangles (NFTs) and hyperphosphorylated tau in the hippocampus and frontal cortex [136]. This tau formation increases with age. In another study, they found that the accumulation of NFTs occurs progressively in the amygdala, hippocampus, brainstem, neocortex, and spinal cord [137]. However, NFTs progressed synapse loss, and decreased synaptic function emerged, with microglial activation preceding the formation of tau tangles. Pre-tangles, characterised by argyrophilic tangle-like inclusions, are displayed in the cortex and hippocampal regions, even in the absence of amyloid pathology [138].

### 3.6. 3xTg-AD

The 3xTg-AD mice model may be beneficial to investigate the emphasis on late-onset AD 3xTg-AD [107]. This mouse model consists of three mutations: APP, PS1, and Tau. Aβ deposition in the 3xTg-AD mouse model begins in the cerebral cortex and subsequently extends to the hippocampus [139]. The administration of RGFP-966, a selective inhibitor of HDAC3, reduces the formation of Aβ1-42 and decreases both tau acetylation and phosphorylation [140]. However, at an intermediate phase of AD, Orta-Salazar et al. [141] showed that the primary motor cortex (M1) region of the 3xTg-AD female mice model had a hyperphosphorylated tau protein and accumulation of Aβ. The development of AD is connected to motor function, which is impacted by the damage of the M1 cell. Changes in synaptic excitability, particularly in hippocampal, can be observed in the 3xTg-AD mice model [142]. These mice models also exhibit episodic memory deficits at 3-6 months of age, which might be caused by the development of an aberrant hyper-excitable state condition in the hippocampal formation rather than a loss of synaptic connectivity. Compared to the female mouse model, male mice exhibit reduced plaque production and no plaques outside the subiculum [107]. In addition, Aβ40 and Aβ42 were additionally elevated in the soluble fractions of the cortical and hippocampal regions of female 3xTg-AD mice, possibly due to enhanced Thy-1 mini-gene. The summary of the mice experimental models of AD is shown in Table 1.

Although existing models have advanced our understanding of human neurodegenerative disease, biomimetic models based on recent technological innovations could further enhance the characterisation of pathological mechanisms [143]. This also makes them more suitable for high-throughput drug screening [144]. Looking ahead, we hope that technological advancements will bring the prospect of personalised medicine closer to reality, even though it is not yet economically feasible.

**Table 1:** Experimental animal models of Alzheimer’s disease.

Transgenic Mouse Model	Neuropathologies
PDAPP	The deposition of Aβ starts to form in the cerebral cortex around six to nine months of age, accompanied by age-related increases in gliosis [109]. Synaptic and dendritic densities in the hippocampal dentate gyrus molecular layer decrease by 8 months of age [109]. Cognitive deficits, particularly memory impairments, become evident by 3 months of age and worsen [109].
5xFAD	High levels of intraneuronal Aβ42 observed [111]. Intracellular staining for Aβ coincided with staining for cathepsin-D [145]. Amyloid pathology in the spinal cord [112]. Neprilysin degrades soluble Aβ peptides [146]. Astrocytic activation is prominent in female mice compared to males [114]. Gliosis and emerging amyloid plaques causing cognitive deficits related to the frontal cortex were observed [113]. Long-term exposure to volatile anaesthetic elevated the Aβ deposition in the hippocampus [118]. Apathy-like behaviour related to soluble Aβ42 and plaques in the hippocampus and prefrontal cortex [105]. Corticolimbic Aβ plaques were the highest detected compared to different models [147]. Calretinin and parvalbumin-positive interneurons are reduced in the hippocampus and dentate gyrus, respectively [119]. Altered glial gap junction expression in the spinal cord was detected [117]. Nurr1 (NR4A2) administration reduced the deposition of Aβ plaques, loss of neuronal, microgliosis, and inhibition of adult hippocampus neurogenesis t [120]. AdipoR2-expressing astrocytes were identified in the dorsomedial hypothalamic and thalamic mediodorsal nuclei [148]. BACE1 increment associated with Aβ overproduction [115]. Mitochondrial dysfunction and cognitive impairment were caused by the buildup of C99 and fl-APP via BACE1-dependent pathways [116].
APP23	Cholinergic deficit in AD observed [126]. Neuron-derived βPP competent inducing amyloid plaque formation and amyloid-associated microglial activation [127]. Aβ deposits were decreased in the absence of enzyme tissue transglutaminase (TG2) [106]. Neuronal subpopulation additions are relatively linked with the expansion of Aβ-deposition [128]. AD patients exhibited notably lower levels of both total and free brain oestrogen (reduced by 60% and 85%, respectively) in comparison to non-AD subjects [129]. Abca1-dependent memory problems in old APP23 mice are probably caused by the quantity of accumulated Aβ oligomers in the hippocampus [125]. Plaques of Aβ deposition in the olfactory nucleus, thalamus, hippocampal, cerebral cortex, and putamen [123]. Selective neuronal death in the brain regions [124]. Increased amyloid plaque accumulation, tau protein hyperphosphorylation, amyloid plaques associated with activated microglia, and pyramidal neuron loss in the CA1 region of the hippocampus [121]. Spatial memory deficits occur before the formation of plaques and the subsequent increase in plaque-associated amyloid-β1–42 peptides [122].
Tg2576	The increase in soluble Aβ levels with age is accompanied by relatively minimal vascular amyloid deposition [131]. Senile plaques disrupt the cortical cytoarchitecture, leading to a gradual loss of antioxidant capacity [134]. Early β-amyloid exposure induced by the upregulation of IL-1β, IL-10, and TGF-βin surrounding reactive astrocytes, both pro- and anti-inflammatory processes [133]. Maintained SYN-IR during ageing is linked to impaired synaptic function, leading to cognitive deficits [132]. Despite the absence of synapse loss, elevated levels of Aβ may be linked to synaptic function and synapse loss [130].
P301S	Progressive accumulation of NFTs occurs in the neocortex, hippocampus, brainstem, amygdala, and spinal cord [137]. Pre-tangles, characterised by argyrophilic tangle-like inclusions observed in the hippocampus and cortex, are present in the lack of amyloid pathology [138]. Tau aggregation initially appears in the hippocampus and cerebral cortex, followed by the presence of NFTs and hyperphosphorylated tau in the frontal cortex and hippocampus, relative to the age-related rise in tau pathology [136]. Hyperphosphorylated tau in the hippocampus and cerebral cortex [135].
3xTg-AD	Aβ deposition initiates in the cerebral cortex and later extends to involve both the hippocampus and cerebral cortex [139]. No plaques are detected outside of the subiculum [107]. Female mice at intermediate disease stages exhibited motor and cellular changes linked to the buildup of Aβ and hyperphosphorylated tau proteins in the M1 region [141]. Reduces buildup of Aβ1–42 and reduces tau phosphorylation and acetylation [140]. Changes in synaptic excitability can be observed [142].

## 4. Biomimetic Approaches in Neurodegenerative Disease Modelling

Developing advanced biomimetic brain environments has become crucial in biomedical research to find effective treatments for AD. Due to AD’s complexity, innovative models that accurately mimic the brain’s intricate microenvironment are needed. These models facilitate better drug screening and the development of targeted therapeutic approaches. Recent scientific efforts have focused on creating biomimetic models designed to replicate the precise complexities of the brain microenvironment. This marks a significant shift in disease modelling and drug screening methodologies.

Computational models, such as the AlzheimerNet model proposed by Shamrat et al. [149], leverage deep learning to classify AD stages based on functional brain alterations in magnetic resonance imaging. This model demonstrates the possibility of artificial intelligence in AD diagnosis and staging by evaluating the performance of models such as VGG16, InceptionV3, AlexNet, MobileNetV2, and ResNet50, with InceptionV3 achieving the most remarkable test accuracy of 96.31% [150,151,152]. Furthermore, data-driven models, such as those developed by Bayraktar et al. [153], utilise molecular communication and artificial neural network models to analyse AD from biomedical and socio-economic perspectives. These models offer innovative approaches to understanding AD progression and diagnosis. Additionally, Gnanadesigan et al. [154] introduced a novel approach that uses intelligent-based deep learning models and network topology measurement to find potential genes for AD. Their study demonstrated the effectiveness of the suggested network topology model for an artificial neural network (ANN) classifier compared to existing models [154].

One notable advancement in this realm is developing a rapid three-dimensional (3D) bioprinting method, which holds immense promise for constructing biomimetic tissue models. Initially applied in replicating glioblastoma microenvironments [155,156,157], this cutting-edge technique exhibits adaptability to the unique challenges posed by AD modelling [158,159]. This introduction sets the stage for exploring the significance of biomimetic brain environments, the latest strides in biomimetic model development, and the potential of 3D bioprinting as a transformative tool for advancing our understanding and treatment options for AD. A 3D-bioprinted vascularised glioblastoma-on-a-chip was suggested to investigate the effects of simulated microgravity, showing promise in developing biomimetic brain microenvironments for disease modelling [160,161]. The study investigated the potential of using camouflaging nanoparticles loaded with brain malignant cancer cell membranes to cross the BBB for imaging and treating brain tumours. This research suggests the possibility of creating targeted drug delivery systems for AD treatment [162,163,164,165].

Recent studies have stressed the significance of incorporating a biomimetic mechanical microenvironment into in vitro models to simulate the tumour microenvironment accurately, which could also be applied to AD modelling [166]. The significance of the microenvironment in AD was emphasised by Qiu et al. [167], who highlighted the molecular and clinical relevance of the brain microenvironment and glycolysis in AD, offering valuable information on potential therapeutic targets. A microfluidic platform is one tool for creating in vitro models that closely mimic the cellular microenvironment and physiological conditions of AD [144]. In AD research, microfluidic platforms are used to investigate various AD mechanisms, including Aβ transmission, Aβ neurotoxicity, Aβ aggregation and clearance, microglial activation, and Tau pathology.

Su et al. [168] proposed that the study on using a nanotheranostic system coated with erythrocyte membrane for targeted immune suppression during AD therapy presents a promising approach for influencing the brain microenvironment in treating AD. Apart from that, astrocytes from individuals with sporadic Alzheimer’s disease (SAD)-linked APOE4 mutations and familial Alzheimer’s disease (FAD)-linked PSEN1M146L mutations showed reduced morphological complexity and changes in the localisation of marker proteins. These findings suggest that FAD and SAD mutations have similar effects on astrocytes [169]. In AD, the multifunctional lipoprotein-biomimetic nanostructure RAP-RL has effectively regulated the cerebral vasculature and re-established the neurovascular unit [170]. RAP-RL consists of an antagonist peptide that binds to the receptor for advanced glycation end-products (RAGE), facilitating the clearance of perivascular Aβ and restoring structure and function within the neurovascular unit. In a study by Ye et al. [171], targeted AD treatment was improved through the synthesis of a macrophage membrane-encapsulated, nitrogen-doped carbon quantum dot (CDQs) nanosystem, which effectively captures excess Cu^2+^ and inhibits rapid Aβ aggregations. Another study utilised red blood cell membranes as templates for the in situ growth of cerium oxide nanocrystals, later encapsulating them with carbon quantum dots to form CDQ-Ce-RBC nanocomposites [172]. When combined with photothermal therapy, this nanocomposite effectively impacts multiple pathways in AD progression The advantage of this approach could be observed through antioxidant protection from CeO_2_ nanocrystals, the inhibition of Aβ aggregation, and the disruption of Aβ fibres.

**Figure 2:**
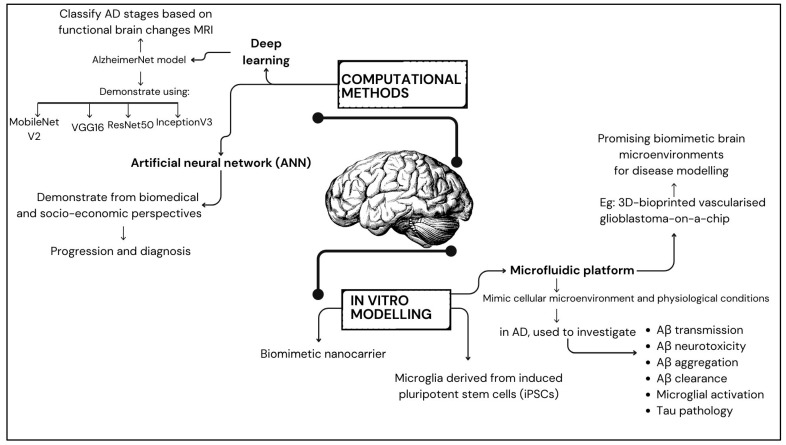
Summary of disease modelling in developing biomimetic brain microenvironment.

Innate immune cells called microglia are found in the brain, carrying out diverse, supportive functions throughout the brain’s development, adulthood, and ageing processes [173]. These functions include synaptic pruning, cellular debris clearance, and neuroinflammation regulation. Microglia generated from healthy patient-derived cells exhibit capabilities such as synaptic pruning, phagocytosis, and Aβ uptake accompanied by the secretion of various cytokines [174]. Exposure to exogenous Aβ triggers alterations in gene expression within these microglia. Conversely, microglia generated from SAD patients created pluripotent stem cells (iPSCs) that exhibit variations in phagocytosis and the increased production of specific cytokines in reaction to lipopolysaccharide treatment [173,174,175]. In addition, biomimetic remodelling of microglial riboflavin metabolism was discovered to alleviate cognitive impairment in AD [176]. This approach involves the design of microglial nanoparticles encapsulating flavin mononucleotides (FMN) for efficient brain delivery [176]. One important enzyme in riboflavin metabolism, riboflavin kinase (RK), is inhibited by the FMN. This inhibition subsequently attenuates the pro-inflammatory TNFR1/NF-Kβ pathway, reducing neuroinflammation (Figure 2).

Another strategy to enhance drug delivery and improve BBB penetration in AD treatments involves the use of biomimetic nanocarriers [177,178]. One example is lipid nanocomposites (APLN), designed to mimic the function and structure of high-density lipoprotein, essential for facilitating microglia-targeted delivery and BBB crossing [177]. These lipid nanocomposites are loaded into methylene blue (MB) to produce APLN/MB, simultaneously targeting both Aβ and Tau pathways. This dual action promotes Aβ clearance by microglia and inhibits Tau phosphorylation, potentially alleviating AD symptoms and improving cognitive functions. In another study, neutrophil membrane-coated metal-organic framework (MOF) nanoenzymes (Neu-MOF/Fla) were used to target inflammatory sites and deliver therapeutic agents [178]. Neu-MOF/Fla is biomimetically engineered to exploit the innate ability of neutrophils to cross the BBB and target inflammatory signals in AD. Solid lipid nanoparticles (SLNs) also show promise as a biomimetic strategy for control nervous system (CNS) drug delivery, enhancing BBB penetration and improving drug bioavailability [179].

## 5. Challenges and Limitations in AD Modelling

Since AD is a complex and complicated disease, modelling it in preclinical research is very challenging. While many current models primarily focus on Aβ and tau pathologies, hallmark features of AD, they often fail to fully capture the condition’s intricacies. One key limitation is the inability to replicate the complex relationship between genetic, environmental, and ageing factors leading to AD pathology [180]. Moreover, neuroinflammation, synaptic dysfunction, and microglial dysregulation, critical components of AD, are frequently overlooked or not accurately represented in existing models. Consequently, these models may not accurately represent the wide range of cellular and molecular alterations seen in AD. Achieving a more comprehensive and accurate representation of AD pathology in preclinical models is vital for advancing our understanding of the disease and creating effective treatment strategies.

Achieving translational relevance in AD modelling poses a formidable challenge for researchers [181]. While animal models provide an essential understanding of the fundamental causes of disease, it can be difficult to apply these discoveries in human clinical trials. The response of animal models to therapeutic interventions often differs from that of humans, creating significant translational barriers [182]. The human brain’s intricate molecular and cellular processes, which are key to disease progression, may be challenging to replicate in animal models accurately. This difficulty hampers the successful transition from promising preclinical results to effective therapeutic strategies for human patients. Therefore, bridging this gap in translational relevance is of utmost importance to ensure that advancements in preclinical AD research can translate into meaningful clinical outcomes [183,184].

The financial burden associated with AD modelling, encompassing the development and maintenance of animal models and conducting extensive preclinical trials, poses a significant challenge. The costs involved in long-term studies, especially those aimed at capturing the gradual progression of AD, can be prohibitive [185,186]. Additionally, the high attrition rates of potential therapies during clinical trials further escalate costs. Despite these challenges, balancing the need for comprehensive and accurate modelling with the economic constraints of research resources remains critical in advancing our understanding and treatment of AD [187].

Scalability is a concern in AD modelling, particularly as research endeavours aim to accommodate the increasing demand for more advanced and high-throughput screening approaches [188,189,190]. Traditional models may need help scaling up efficiently to meet the growing requirements of large-scale studies and drug screening initiatives. Therefore, developing robust and scalable models that include diverse genetic backgrounds and environmental factors is essential to enhancing the efficiency and productivity of AD research. Overcoming scalability challenges is pivotal for the field to accommodate the expanding breadth of investigations needed for a comprehensive understanding of AD pathogenesis and treatment development.

## 6. Conclusions

AD presents a significant global health challenge, requiring innovative approaches for advancing models and treatments. This comprehensive review highlights the systematic enhancement of biomimetic brain environments to address limitations in current AD models and enhance the effectiveness of drug screening. Integrating biomolecular components such as Aβ plaques and tau proteins would improve the dependability in representing disease mechanisms. Expected outcomes include establishing a robust platform for high-throughput screening with enhanced scalability and translational relevance, potentially accelerating drug discovery. This study addresses gaps in AD modelling and demonstrates the potential for refining precise and effective drug treatments.
